# Poly[[tetra­aqua­(μ_6_-2,2′-diiodo­biphenyl-4,4′,5,5′-tetra­carboxyl­ato)dizinc(II)] dihydrate]

**DOI:** 10.1107/S1600536808026494

**Published:** 2008-08-30

**Authors:** Yan Wang, Yue-Qin Li, Ying-Zhong Shen

**Affiliations:** a Applied Chemistry Department, School of Material Science & Engineering, Nanjing University of Aeronautics & Astronautics, Nanjing 210016, People’s Republic of China

## Abstract

In the title compound, {[Zn_2_(C_16_H_4_I_2_O_8_)(H_2_O)_4_]·2H_2_O}_*n*_, two crystallographically independent Zn^II^ atoms are each located on a twofold rotation axis. Both Zn^II^ atoms are in distorted octa­hedral coordination geometries: one is coordinated by six O atoms from four carboxyl­ate groups, while the other is coordinated by two carboxyl­ate groups and four water mol­ecules. The tetra­carboxyl­ate ligand mol­ecules connect the Zn^II^ atoms, completing a three-dimensional metal–organic framework. O—H⋯O hydrogen bonds link the metal–organic framework with the uncoord­inated water mol­ecules.

## Related literature

For related literature, see: Beringer *et al.* (1953 ▶); Cordes *et al.* (2006 ▶); Garay *et al.* (2007 ▶); Noro *et al.* (2007 ▶); Qiu *et al.* (2007 ▶); Wang *et al.* (2007 ▶); Weng *et al.* (2007 ▶); Williams *et al.* (2005 ▶).
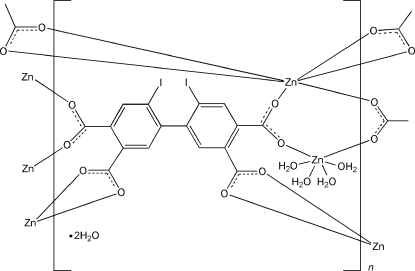


## Experimental

### 

#### Crystal data

[Zn_2_(C_16_H_4_I_2_O_8_)(H_2_O)_4_]·2H_2_O
*M* *_r_* = 816.83
Monoclinic, 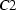

*a* = 10.9466 (16) Å
*b* = 9.8135 (14) Å
*c* = 11.3913 (17) Å
β = 100.187 (3)°
*V* = 1204.4 (3) Å^3^
*Z* = 2
Mo *K*α radiation
μ = 4.62 mm^−1^
*T* = 295 (2) K
0.40 × 0.30 × 0.20 mm

#### Data collection

Bruker SMART APEX CCD area-detector diffractometer
Absorption correction: numerical (**SADABS**; Bruker, 2000 ▶) *T*_min_ = 0.20, *T*_max_ = 0.39
3279 measured reflections
2126 independent reflections
1767 reflections with *I* > 2σ(*I*)
*R*_int_ = 0.040

#### Refinement

*R*[*F* ^2^ > 2σ(*F* ^2^)] = 0.052
*wR*(*F* ^2^) = 0.088
*S* = 0.97
2126 reflections
155 parameters
1 restraint
H-atom parameters constrained
Δρ_max_ = 1.07 e Å^−3^
Δρ_min_ = −1.01 e Å^−3^
Absolute structure: Flack (1983 ▶), 870 Friedel pairs
Flack parameter: 0.00 (4)

### 

Data collection: *SMART* (Bruker, 2000 ▶); cell refinement: *SAINT* (Bruker, 2000 ▶); data reduction: *SAINT*; program(s) used to solve structure: *SHELXS97* (Sheldrick, 2008 ▶); program(s) used to refine structure: *SHELXL97* (Sheldrick, 2008 ▶); molecular graphics: *SHELXTL* (Sheldrick, 2008 ▶); software used to prepare material for publication: *SHELXTL*.

## Supplementary Material

Crystal structure: contains datablocks I, global. DOI: 10.1107/S1600536808026494/is2308sup1.cif

Structure factors: contains datablocks I. DOI: 10.1107/S1600536808026494/is2308Isup2.hkl

Additional supplementary materials:  crystallographic information; 3D view; checkCIF report

## Figures and Tables

**Table 1:** Hydrogen-bond geometry (Å, °)

*D*—H⋯*A*	*D*—H	H⋯*A*	*D*⋯*A*	*D*—H⋯*A*
O5—H5*A*⋯O3^i^	0.85	2.35	3.074 (9)	144
O5—H5*A*⋯O1^ii^	0.85	2.30	2.967 (9)	136
O5—H5*B*⋯O7^iii^	0.85	1.97	2.807 (15)	169
O6—H6*A*⋯O3^iv^	0.85	2.01	2.772 (10)	148
O6—H6*B*⋯O7	0.85	2.50	3.113 (16)	129
O7—H7*B*⋯O4	0.85	2.23	2.910 (16)	137

Symmetry codes: (i) 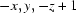; (ii) 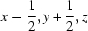; (iii) 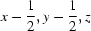; (iv) 
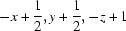
.

**Fig. 1.**
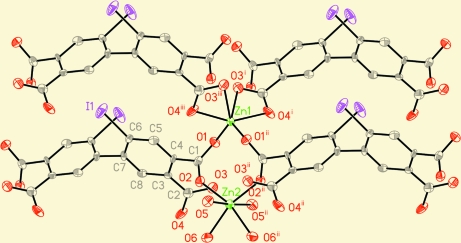
A view of the title complex, depicting the ZnII coordination environment. Displacement ellipsoids are drawn at the 30% probability level. The solvent water molecules and H atoms have been omitted for clarity [symmetry codes: (i) -x + 1/2, y - 1/2, -z + 1; (ii) -x, y, -z + 1; (iii) x - 1/2, y - 1/2, z].

**Fig. 2.**
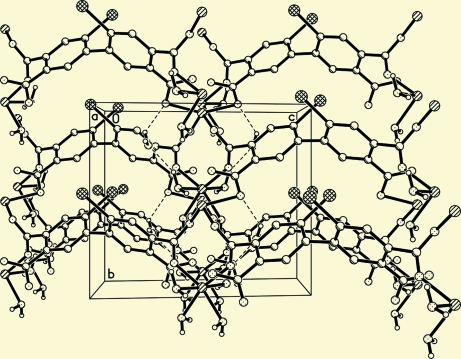
Packing diagram of the title complex viewed along the a axis, showing a three-dimensional metal-organic framework. The dashed lines indicate hydrogen bonds.

## supplementary crystallographic information

### Comment

Interest in the self-assembled construction of coordination polymers is rapidly increasing not only owing to their potential applications in gas storage, ion-exchange, catalysis, electrical conductivity, nonlinear optics and magnetism, but also because of their fascinating diversified architectures and topologies (Cordes *et al.*, 2006; Garay *et al.*, 2007). Multicarboxylate ligands, such as 1,4-benzenedicarboxylate (Williams *et al.*, 2005), 1,3,5-benzenetricarboxylate (Noro *et al.*, 2007) and biphenyl-3,3',4,4'-tetracarboxylate (Weng *et al.*, 2007), have been extensively employed in the construction of novel metal-organic complexes with multidimensional networks and interesting properties. In view of the excellent coordination capability of multicarboxylate anions and the solubility of diaryliodonium salts, we employed 2,2'-diiodobiphenyl-4,4',5,5'-tetracarboxylic acid (H_4_*L*), as an organic building unit to generate three dimensional metal-organic framework. In this paper, we describe the synthesis of a novel zinc(II) complexes, namely, [Zn_2_(*L*)(H_2_O)_4._2H_2_O]*_n_*, by reaction of Zn(NO_3_)_2_.6H_2_O and H_4_*L via* hydrothermal method, which was characterized by IR, elemental analysis and X-ray single-crystal analysis. To the best of our knowledge, transition metal coordination polymers based on diiodobiphenyl tetracarboxylate has never been reported before. This work may provide useful information for the further design of metal-organic frameworks with interesting architectures using diiodobiphenyl tetracarboxylate as versatile multidentate ligands.

The title complex crystallizes in the monoclinic system, space group *C*2, with two crystallographically independent Zn^II^ atoms each located on a twofold axis. As shown in Fig. 1, both Zn^II^ atoms are in distorted octahedral configurations and are connected by carboxylate groups. The Zn1 center is coordinated by four carboxylate groups, that is, 4(4')-COO^-^ from two different *L*^4-^ ligands in a monodentate fashion (O1 and O1^ii^) and 5(5')-COO^-^ from other two *L*^4-^ ligands in a bischelating fashion (O3^i^, O4^i^, O3^iii^ and O4^iii^). In contrast, the Zn2 center is coordinated by four O atoms of water (O5, O6, O5^ii^ and O6^ii^) and two carboxylate groups: 4(4')-COO^-^ from two different *L*^4-^ ligands in a monodentate fashion (O2 and O2^ii^). The Zn–O bond lengths fall in the range of 1.953 (6)–2.368 (7) Å, similar to those in other zinc-tetracarboxylate coordination polymers (Wang *et al.*, 2007). Hence, the *L*^4-^ ligand acts as a octadentate ligand, linking six different Zn^II^ atoms to form a three-dimensional metal–organic framework (Fig. 2); the 5,5'-carboxyl groups adopt a bidentate bridging mode, while the 4,4'-carboxyl groups exhibit a bis(monodentate) bridging mode. Within the *L*^4-^ ligand, the two phenyl rings are almost perpendicular to each other with the dihedral angle of 88.6 (1)°, and the dihedral angles between 4,5(4',5')-carboxylate groups and the plane of correspondingly linked phenyl rings are respectively 72.0 (1) and 27.3 (1)°.

There are various O—H···O hydrogen bonds associated with the coordinated water molecules, uncoordinated water molecules and carboxylate O atoms in the title complex, linking the metal-organic framework with the uncoordinated water molecules.

### Experimental

All chemicals were of analytical grade and used without further purification. According to the reported procedure (Beringer *et al.*, 1953; Qiu *et al.*, 2007), H_4_*L* was prepared by iodine substitution of the *N*-methyl protected 3,3',4,4'-biphenyltetracarboxylicdianhydride, hydrolysis by 3*M* KOH and acidification by 6.5 *M* HCl to pH 1.0. The hydrothermal reaction was performed in a 25 ml Teflon-lined stainless steel autoclave under autogenous pressure. An solution of H_4_*L* (58 mg, 0.1 mmol), Zn(NO_3_)_2_.6H_2_O (59 mg, 0.2 mmol), 2 ml EtOH and 8 ml H_2_O was adjusted to pH 7 with 1 *M* NaOH solution and then heated at 140 °C for 3 days. After the sample was cooled slowly to room temperature, colourless crystals were obtained and air-dried by filtration *ca* 60% yield based on Zn. Anal. calcd for C_8_H_8_O_7_IZn: C 23.53, H 1.97%; Found: C 23.48, H 2.02%. IR (KBr discs, *ν*_max_/cm^-1^): 3379(*vs*), 1599(*s*), 1562(*s*), 1539(*s*), 1472(*w*), 1401(*vs*), 1318(*w*), 1254(*m*), 1163(*w*), 1098(*m*), 953(*w*), 915(*w*), 873(*m*), 846(*m*), 802(*m*), 678(*w*), 605(*w*), 543(*w*).

### Refinement

O-bound H atoms were located in a difference Fourier map and then constrained to ride on their parent atoms, with O—H = 0.85 Å and with *U*_iso_(H) = 1.2*U*_eq_(O). Other H atoms were positioned geometrically (C—H = 0.93 Å) and constrained to ride on their parent atoms, with *U*_iso_(H) = 1.2*U*_eq_(C). The highest peak and the deepest hole are 0.34 and 0.97 Å, respectively, from I1.

### Crystal data

**Table:**

[Zn_2_(C_16_H_4_I_2_O_8_)(H_2_O)_4_]·2H_2_O	*F*_000_ = 780
*M**_r_* = 816.83	*D*_x_ = 2.252 Mg m^−^^3^
Monoclinic, *C*2	Mo *K*α radiation λ = 0.71069 Å
Hall symbol: C 2y	Cell parameters from 762 reflections
*a* = 10.9466 (16) Å	θ = 2.8–18.3º
*b* = 9.8135 (14) Å	µ = 4.62 mm^−^^1^
*c* = 11.3913 (17) Å	*T* = 295 (2) K
β = 100.187 (3)º	Block, colourless
*V* = 1204.4 (3) Å^3^	0.40 × 0.30 × 0.20 mm
*Z* = 2	

### Data collection

**Table:**

Bruker SMART APEX CCD area-detector diffractometer	2126 independent reflections
Radiation source: fine-focus sealed tube	1767 reflections with *I* > 2σ(*I*)
Monochromator: graphite	*R*_int_ = 0.040
*T* = 295(2) K	θ_max_ = 26.0º
φ and ω scans	θ_min_ = 2.8º
Absorption correction: numerical(*SADABS*; Bruker, 2000)	*h* = −9→13
*T*_min_ = 0.20, *T*_max_ = 0.39	*k* = −11→12
3279 measured reflections	*l* = −14→14

### Refinement

**Table:**

Refinement on *F*^2^	Hydrogen site location: inferred from neighbouring sites
Least-squares matrix: full	H-atom parameters constrained
*R*[*F*^2^ > 2σ(*F*^2^)] = 0.052	*w* = 1/[σ^2^(*F*_o_^2^) + (0.0207*P*)^2^] where *P* = (*F*_o_^2^ + 2*F*_c_^2^)/3
*wR*(*F*^2^) = 0.088	(Δ/σ)_max_ < 0.001
*S* = 0.98	Δρ_max_ = 1.07 e Å^−^^3^
2126 reflections	Δρ_min_ = −1.01 e Å^−^^3^
155 parameters	Extinction correction: none
1 restraint	Absolute structure: Flack (1983), 870 Friedel pairs
Primary atom site location: structure-invariant direct methods	Flack parameter: 0.00 (4)
Secondary atom site location: difference Fourier map	

### Special details

**Table:**

Geometry. All e.s.d.'s (except the e.s.d. in the dihedral angle between two l.s. planes) are estimated using the full covariance matrix. The cell e.s.d.'s are taken into account individually in the estimation of e.s.d.'s in distances, angles and torsion angles; correlations between e.s.d.'s in cell parameters are only used when they are defined by crystal symmetry. An approximate (isotropic) treatment of cell e.s.d.'s is used for estimating e.s.d.'s involving l.s. planes.
Refinement. Refinement of *F*^2^ against ALL reflections. The weighted *R*-factor *wR* and goodness of fit *S* are based on *F*^2^, conventional *R*-factors *R* are based on *F*, with *F* set to zero for negative *F*^2^. The threshold expression of *F*^2^ > σ(*F*^2^) is used only for calculating *R*-factors(gt) *etc*. and is not relevant to the choice of reflections for refinement. *R*-factors based on *F*^2^ are statistically about twice as large as those based on *F*, and *R*- factors based on ALL data will be even larger.

### Fractional atomic coordinates and isotropic or equivalent isotropic displacement parameters (Å^2^)

**Table:**

	*x*	*y*	*z*	*U*_iso_*/*U*_eq_	
I1	0.31728 (8)	0.46912 (8)	1.05780 (7)	0.0770 (3)	
Zn1	0.0000	0.52673 (14)	0.5000	0.0296 (4)	
Zn2	0.0000	0.94976 (18)	0.5000	0.0373 (4)	
O1	0.1541 (6)	0.6253 (7)	0.5567 (6)	0.0404 (17)	
O2	0.1099 (7)	0.8284 (7)	0.6272 (6)	0.0399 (18)	
O3	0.3711 (6)	0.8475 (7)	0.5451 (6)	0.0454 (19)	
O4	0.4839 (6)	0.9757 (8)	0.6747 (6)	0.0543 (19)	
O5	−0.1277 (5)	0.9419 (7)	0.6112 (5)	0.0454 (18)	
H5A	−0.2029	0.9518	0.5787	0.054*	
H5B	−0.1254	0.8743	0.6577	0.054*	
O6	0.1005 (8)	1.1179 (7)	0.5892 (7)	0.057 (2)	
H6A	0.1232	1.1982	0.5746	0.068*	
H6B	0.1538	1.0920	0.6488	0.068*	
O7	0.3492 (12)	1.2077 (15)	0.7470 (11)	0.116 (4)	
H7A	0.3346	1.2367	0.8145	0.139*	
H7B	0.4010	1.1431	0.7670	0.139*	
C1	0.1734 (8)	0.7220 (12)	0.6301 (8)	0.032 (2)	
C2	0.4137 (9)	0.8752 (11)	0.6505 (9)	0.038 (3)	
C3	0.3828 (10)	0.7879 (9)	0.7465 (9)	0.030 (2)	
C4	0.2774 (9)	0.7090 (10)	0.7331 (8)	0.027 (2)	
C5	0.2594 (9)	0.6187 (9)	0.8224 (9)	0.036 (2)	
H5	0.1881	0.5652	0.8124	0.043*	
C6	0.3468 (9)	0.6073 (9)	0.9266 (8)	0.033 (2)	
C7	0.4536 (8)	0.6899 (9)	0.9425 (7)	0.028 (2)	
C8	0.4700 (9)	0.7767 (9)	0.8536 (9)	0.031 (2)	
H8	0.5411	0.8305	0.8636	0.038*	

### Atomic displacement parameters (Å^2^)

**Table:**

	*U*^11^	*U*^22^	*U*^33^	*U*^12^	*U*^13^	*U*^23^
I1	0.0650 (6)	0.0980 (7)	0.0621 (5)	−0.0337 (6)	−0.0053 (4)	0.0436 (5)
Zn1	0.0288 (9)	0.0325 (9)	0.0269 (8)	0.000	0.0029 (7)	0.000
Zn2	0.0294 (8)	0.0396 (10)	0.0414 (9)	0.000	0.0021 (7)	0.000
O1	0.030 (4)	0.042 (4)	0.047 (5)	0.000 (4)	0.000 (3)	−0.011 (3)
O2	0.037 (4)	0.049 (5)	0.032 (4)	0.015 (4)	−0.001 (3)	0.001 (3)
O3	0.049 (5)	0.050 (5)	0.040 (4)	0.009 (4)	0.015 (4)	0.004 (4)
O4	0.041 (4)	0.054 (5)	0.062 (5)	−0.014 (4)	−0.006 (3)	0.032 (4)
O5	0.027 (3)	0.057 (5)	0.053 (4)	0.012 (4)	0.009 (3)	0.009 (4)
O6	0.079 (6)	0.035 (4)	0.048 (5)	−0.012 (4)	−0.010 (4)	0.010 (3)
O7	0.118 (10)	0.116 (9)	0.124 (10)	0.014 (8)	0.049 (8)	0.031 (8)
C1	0.028 (5)	0.039 (6)	0.029 (5)	−0.006 (5)	0.008 (4)	0.009 (5)
C2	0.027 (6)	0.041 (7)	0.043 (7)	0.004 (5)	−0.003 (5)	0.008 (5)
C3	0.035 (6)	0.021 (5)	0.033 (6)	−0.005 (4)	0.006 (5)	−0.002 (4)
C4	0.035 (6)	0.029 (5)	0.017 (5)	0.006 (5)	0.000 (4)	0.008 (4)
C5	0.026 (6)	0.035 (6)	0.047 (7)	−0.004 (5)	0.009 (5)	0.001 (5)
C6	0.039 (6)	0.028 (5)	0.028 (6)	0.007 (5)	−0.002 (5)	0.003 (4)
C7	0.023 (5)	0.042 (6)	0.022 (5)	0.007 (4)	0.007 (4)	−0.006 (4)
C8	0.021 (5)	0.030 (6)	0.043 (6)	−0.008 (4)	0.006 (5)	0.001 (4)

### Geometric parameters (Å, °)

**Table:**

I1—C6	2.085 (9)	O5—H5A	0.85
Zn1—O1	1.953 (6)	O5—H5B	0.85
Zn1—O1^i^	1.953 (6)	O6—H6A	0.85
Zn1—O4^ii^	2.090 (6)	O6—H6B	0.85
Zn1—O4^iii^	2.090 (6)	O7—H7A	0.85
Zn1—O3^iii^	2.368 (7)	O7—H7B	0.85
Zn1—O3^ii^	2.368 (7)	C1—C4	1.488 (12)
Zn2—O5	2.047 (5)	C2—C3	1.475 (13)
Zn2—O5^i^	2.047 (5)	C3—C4	1.375 (13)
Zn2—O2^i^	2.085 (7)	C3—C8	1.415 (14)
Zn2—O2	2.085 (7)	C4—C5	1.389 (12)
Zn2—O6	2.138 (8)	C5—C6	1.391 (13)
Zn2—O6^i^	2.138 (7)	C5—H5	0.9300
O1—C1	1.258 (12)	C6—C7	1.408 (12)
O2—C1	1.251 (12)	C7—C8	1.359 (12)
O3—C2	1.239 (11)	C7—C7^iv^	1.509 (17)
O4—C2	1.251 (11)	C8—H8	0.9300
			
O1—Zn1—O1^i^	120.6 (4)	C1—O2—Zn2	138.1 (6)
O1—Zn1—O4^ii^	102.7 (3)	C2—O3—Zn1^v^	84.9 (6)
O1^i^—Zn1—O4^ii^	91.0 (3)	C2—O4—Zn1^v^	97.5 (6)
O1—Zn1—O4^iii^	91.0 (3)	H5A—O5—H5B	106.00
O1^i^—Zn1—O4^iii^	102.7 (3)	H6A—O6—H6B	104.00
O4^ii^—Zn1—O4^iii^	152.3 (5)	H7A—O7—H7B	103.00
O1—Zn1—O3^iii^	144.0 (3)	O2—C1—O1	125.7 (9)
O1^i^—Zn1—O3^iii^	85.8 (2)	O2—C1—C4	115.9 (9)
O4^ii^—Zn1—O3^iii^	100.5 (3)	O1—C1—C4	118.3 (10)
O4^iii^—Zn1—O3^iii^	57.4 (3)	O3—C2—O4	119.9 (10)
O1—Zn1—O3^ii^	85.8 (2)	O3—C2—C3	119.6 (9)
O1^i^—Zn1—O3^ii^	144.0 (3)	O4—C2—C3	120.5 (9)
O4^ii^—Zn1—O3^ii^	57.4 (3)	C4—C3—C8	118.5 (8)
O4^iii^—Zn1—O3^ii^	100.5 (3)	C4—C3—C2	122.9 (9)
O3^iii^—Zn1—O3^ii^	84.1 (3)	C8—C3—C2	118.4 (9)
O5—Zn2—O5^i^	175.7 (4)	C3—C4—C5	120.2 (9)
O5—Zn2—O2^i^	92.0 (3)	C3—C4—C1	123.3 (8)
O5^i^—Zn2—O2^i^	85.5 (3)	C5—C4—C1	116.3 (9)
O5—Zn2—O2	85.5 (3)	C4—C5—C6	120.7 (9)
O5^i^—Zn2—O2	92.0 (3)	C4—C5—H5	120
O2^i^—Zn2—O2	110.3 (4)	C6—C5—H5	120
O5—Zn2—O6	94.9 (3)	C5—C6—C7	119.6 (9)
O5^i^—Zn2—O6	88.4 (3)	C5—C6—I1	119.6 (7)
O2^i^—Zn2—O6	163.0 (3)	C7—C6—I1	120.8 (7)
O2—Zn2—O6	85.7 (3)	C8—C7—C6	118.8 (9)
O5—Zn2—O6^i^	88.4 (3)	C8—C7—C7^iv^	119.3 (9)
O5^i^—Zn2—O6^i^	94.9 (3)	C6—C7—C7^iv^	121.9 (8)
O2^i^—Zn2—O6^i^	85.7 (3)	C7—C8—C3	122.2 (9)
O2—Zn2—O6^i^	163.0 (3)	C7—C8—H8	119
O6—Zn2—O6^i^	79.0 (4)	C3—C8—H8	119
C1—O1—Zn1	128.9 (6)		
			
O1^i^—Zn1—O1—C1	49.0 (8)	O3—C2—C3—C8	150.4 (9)
O4^ii^—Zn1—O1—C1	147.9 (8)	O4—C2—C3—C8	−28.7 (14)
O4^iii^—Zn1—O1—C1	−56.4 (9)	C8—C3—C4—C5	−1.3 (16)
O3^iii^—Zn1—O1—C1	−83.0 (9)	C2—C3—C4—C5	173.6 (8)
O3^ii^—Zn1—O1—C1	−156.9 (8)	C8—C3—C4—C1	173.6 (9)
C2^iii^—Zn1—O1—C1	−67.9 (9)	C2—C3—C4—C1	−11.5 (17)
C2^ii^—Zn1—O1—C1	176.4 (8)	O2—C1—C4—C3	−70.1 (13)
O5—Zn2—O2—C1	132.2 (10)	O1—C1—C4—C3	112.1 (12)
O5^i^—Zn2—O2—C1	−44.3 (10)	O2—C1—C4—C5	105.0 (11)
O2^i^—Zn2—O2—C1	41.7 (9)	O1—C1—C4—C5	−72.8 (12)
O6—Zn2—O2—C1	−132.6 (11)	C3—C4—C5—C6	0.4 (15)
O6^i^—Zn2—O2—C1	−158.2 (10)	C1—C4—C5—C6	−174.8 (9)
Zn2—O2—C1—O1	−28.8 (16)	C4—C5—C6—C7	1.1 (14)
Zn2—O2—C1—C4	153.7 (7)	C4—C5—C6—I1	−179.4 (7)
Zn1—O1—C1—O2	−50.1 (14)	C5—C6—C7—C8	−1.7 (13)
Zn1—O1—C1—C4	127.4 (8)	I1—C6—C7—C8	178.8 (6)
Zn1^v^—O3—C2—O4	5.3 (9)	C5—C6—C7—C7^iv^	175.1 (8)
Zn1^v^—O3—C2—C3	−173.8 (9)	I1—C6—C7—C7^iv^	−4.4 (11)
Zn1^v^—O4—C2—O3	−6.1 (10)	C6—C7—C8—C3	0.8 (13)
Zn1^v^—O4—C2—C3	173.0 (8)	C7^iv^—C7—C8—C3	−176.1 (9)
O3—C2—C3—C4	−24.4 (15)	C4—C3—C8—C7	0.7 (16)
O4—C2—C3—C4	156.5 (10)	C2—C3—C8—C7	−174.4 (8)

Symmetry codes: (i) −*x*, *y*, −*z*+1; (ii) −*x*+1/2, *y*−1/2, −*z*+1; (iii) *x*−1/2, *y*−1/2, *z*; (iv) −*x*+1, *y*, −*z*+2; (v) *x*+1/2, *y*+1/2, *z*.

### Hydrogen-bond geometry (Å, °)

**Table:**

*D*—H···*A*	*D*—H	H···*A*	*D*···*A*	*D*—H···*A*
O5—H5A···O3^i^	0.85	2.35	3.074 (9)	144
O5—H5A···O1^vi^	0.85	2.30	2.967 (9)	136
O5—H5B···O7^iii^	0.85	1.97	2.807 (15)	169
O6—H6A···O3^vii^	0.85	2.01	2.772 (10)	148
O6—H6B···O7	0.85	2.50	3.113 (16)	129
O7—H7B···O4	0.85	2.23	2.910 (16)	137

Symmetry codes: (i) −*x*, *y*, −*z*+1; (vi) *x*−1/2, *y*+1/2, *z*; (iii) *x*−1/2, *y*−1/2, *z*; (vii) −*x*+1/2, *y*+1/2, −*z*+1.
